# Targeting the Infant Gut Microbiota Through a Perinatal Educational Dietary Intervention: Protocol for a Randomized Controlled Trial

**DOI:** 10.2196/14771

**Published:** 2019-10-21

**Authors:** Samantha L Dawson, Jeffrey M Craig, Gerard Clarke, Mohammadreza Mohebbi, Phillip Dawson, Mimi LK Tang, Felice N Jacka

**Affiliations:** 1 Food & Mood Centre, IMPACT Strategic Research Centre Deakin University Geelong Australia; 2 Environmental & Genetic Epidemiology Research Murdoch Children’s Research Institute Royal Children’s Hospital Parkville Australia; 3 School of Medicine Deakin University Waurn Ponds Australia; 4 Department of Psychiatry and Neurobehavioural Science University College Cork Cork Ireland; 5 APC Microbiome Ireland University College Cork Cork Ireland; 6 INFANT Research Centre University College Cork Cork Ireland; 7 Biostatistics Unit, Faculty of Health Deakin University Geelong Australia; 8 Centre for Research in Assessment and Digital Learning Deakin University Melbourne Australia; 9 Allergy and Immune Disorders Murdoch Children’s Research Institute Royal Children’s Hospital Parkville Australia; 10 Department of Paediatrics University of Melbourne Parkville Australia; 11 Centre for Adolescent Health Murdoch Children’s Research Institute Royal Children’s Hospital Parkville Australia; 12 Black Dog Institute New South Wales Australia

**Keywords:** gastrointestinal microbiome, diet, pregnancy, infant, newborn, randomized controlled trial

## Abstract

**Background:**

The early life gut microbiota are an important regulator of the biological pathways contributing toward the pathogenesis of noncommunicable disease. It is unclear whether improvements to perinatal diet quality could alter the infant gut microbiota.

**Objective:**

The aim of this study is to assess the efficacy of a perinatal educational dietary intervention in influencing gut microbiota in mothers and infants 4 weeks after birth.

**Methods:**

The Healthy Parents, Healthy Kids randomized controlled trial aimed to recruit 90 pregnant women from Melbourne, Victoria, Australia. At week 26 of gestation, women were randomized to receive dietary advice from their doctor (n=45), or additionally receive a dietary intervention (n=45). The intervention included an educational workshop and 2 support calls aiming to align participants’ diets with the Australian Dietary Guidelines and increase intakes of prebiotic and probiotic foods. The educational design focused on active learning and self-assessment. Behavior change techniques were used to support dietary adherence, and the target behavior was eating for the gut microbiota. Exclusion criteria were age under 18 years, diagnosed mental illnesses, obesity, diabetes mellitus, diagnosed bowel conditions, exclusion diets, illicit drug use, antibiotic use, prebiotic or probiotic supplementation, and those lacking dietary autonomy. The primary outcome measure is a between-group difference in alpha diversity in infant stool collected 4 weeks after birth. Secondary outcomes include evaluating the efficacy of the intervention in influencing infant and maternal stool microbial composition and short chain fatty acid concentrations, epigenetic profile, and markers of inflammation and stress, as well as changes in maternal dietary intake and well-being. The study and intervention feasibility and acceptance will also be evaluated as secondary outcomes.

**Results:**

The study results are yet to be written. The first participant was enrolled on July 28, 2016, and the final follow-up assessment was completed on October 11, 2017.

**Conclusions:**

Data from this study will provide new insights regarding the ability of interventions targeting the perinatal diet to alter the maternal and infant gut microbiota. If this intervention is proven, our findings will support larger studies aiming to guide the assembly of gut microbiota in early life.

**Trial Registration:**

Australian Clinical Trials Registration Number ACTRN12616000936426; https://www.anzctr.org.au/Trial/Registration/TrialReview.aspx?id=370939

**International Registered Report Identifier (IRRID):**

DERR1-10.2196/14771

## Introduction

### Background

The diversity and composition of the neonatal gut microbiota is garnering interest as a target for the prevention of noncommunicable diseases. The disappearing microbiome hypothesis contends that reduced bacterial diversity over generations results in increased allergic and metabolic disease risk in children [1]. In 1-month old infants, low microbial diversity is associated with an increased risk of later atopic eczema [2], allergic sensitization, allergic rhinitis, peripheral blood eosinophilia [3], and asthma [4]. In addition, differential microbial composition is associated with an increased risk of noncommunicable diseases, including allergic sensitization [3], eczema [2], asthma risk [5], neurodevelopmental outcomes [6], and later adiposity in infants [7]. Hence, novel methods of altering the neonatal gut microbiome are of interest. The influence of poor maternal diet (high fat or low fiber) on offspring gut microbiota has been studied in animals, demonstrating that poor maternal diets disturb offspring gut microbiota [8,9]. To our knowledge, there are no human randomized controlled trials (RCTs) with the primary aim of testing whether the maternal diet can modify the diversity and composition of the infant gut microbiota. Dietary supplementation trials of perinatal prebiotic or probiotic supplements provide a premise for testing this aim, with some studies indicating that these supplements modify the composition of gut microbiota in mothers [10] and infants [11,12]. Importantly, though, a supplementation approach fails to address the quality of the underlying diet. In humans, the prenatal diet has been associated with the composition of the infant gut microbiota [13,14], but it is still unclear whether this relationship is modifiable. Human studies are needed to determine whether infant gut microbiota can be modified through perinatal dietary change.

In murine [8] and primate [9] models, poor-quality prenatal diets disturb vertical transmission of microbiota (from mother to offspring). For example, a prenatal diet devoid of dietary fiber reduced microbial diversity and the abundance of fiber-degrading taxa in mothers and offspring [8]. Low diversity compounded over 4 generations and could not be corrected via a high-fiber diet. Similarly, compared with a low-fat prenatal diet (13% of energy from soya bean oil), a high-fat prenatal diet altered the microbiota of vaginally born primates [9]. This alteration was persistent at 1 year and could not be corrected by weaning offspring onto a low-fat diet [9]. In humans, compared with a low-fat prenatal diet (24% of daily energy from fat), a high-fat (43%) diet during pregnancy was associated with an altered infant gut microbiome, including a depletion of *Bacteroides* persisting to 4 to 6 weeks of age [13]. Taken together, these results suggest that poor-quality diets (such as low fiber, high saturated fatty acid, and high sugar content) during pregnancy and lactation disturb vertical transmission. However, a causal relationship between the maternal diet and neonatal microbial acquisition is yet to be established in humans.

Healthy dietary patterns that are high in fiber and low in fat are associated with higher microbial alpha diversity in adults [15]. Population-based metagenomic analysis indicates that the dietary features that are associated with higher alpha diversity (as measured by the Shannon Index) are frequent fruit and vegetable consumption along with polyphenol-containing tea, coffee, and red wine [16]. Conversely, dietary features associated with low alpha diversity are sugar-sweetened soda, whole fat milk, savory snacking, and a high total energy intake. Across the developed countries, the mean daily intake of fiber for pregnant women is 18 (SD 4.4) g, this is below the recommended ranges (21-28 g depending on country) [17]. Similarly, mean saturated fat intakes of 32.2 (SD 9.1) g/day were 8.5% to 16.5% above the recommended ranges (depending on country) [17]. In Australia, pregnant women have poor diet quality; they neither know nor meet the Dietary Guidelines for all 5 food groups [18-20], but they are motivated and would like further nutritional education [18].

### Objectives

The Healthy Parents, Healthy Kids (HPHK) study (Trial registration: Australian New Zealand Clinical Trials Registry, ACTRN12616000936426) is a prospectively registered open-label, parallel group, RCT of an educational perinatal dietary intervention targeting gut microbiota from the third trimester of pregnancy until 4 weeks after birth. The primary aim is to evaluate whether the dietary intervention alters alpha diversity of the infant gut microbiota 4 weeks after birth. Secondary aims are to evaluate the efficacy of the intervention in altering microbiota, inflammatory and stress profiles, epigenetic regulation, and maternal diet and well-being. The feasibility and acceptability of the study intervention will also be evaluated. The HPHK study intervention design couples pedagogical theory and educational design (focused on self-assessment and self-efficacy) with Behavior Change Techniques (BCTs) [21] to support efficacy and dietary adherence. A sound educational design is an important, yet seemingly overlooked consideration; first, it ensures that participants are able to *do* the target behavior, second, it helps to mitigate against confusing a true null effect with insufficient learning, and third, it safeguards the literature against spurious findings from poorly designed interventions.

## Methods

**Figure 1:**
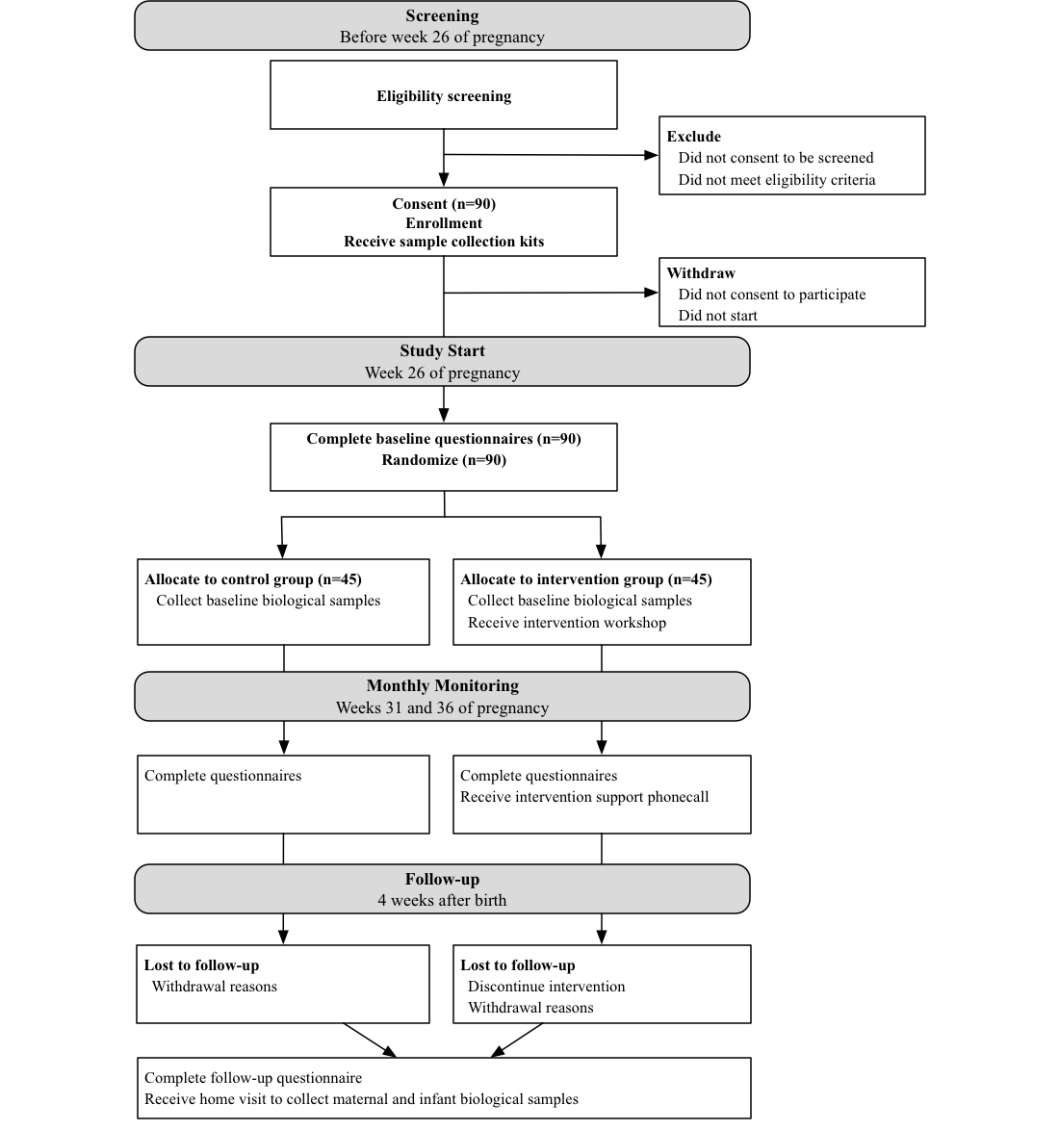
Study flow diagram. Displays the timing for each activity of the randomized controlled trial.

This protocol was written according to the SPIRIT 2013 statement [22]. See Figure 1 for the study flow diagram.

### Primary Hypothesis

The dietary intervention will result in increased microbial diversity (Shannon index) in infants measured 4 weeks after birth, compared with the control group.

### Secondary Hypotheses

#### Microbiota

Using stool samples collected at follow-up, compared with the control group, the intervention group will have (1) dissimilarity in infant stool operational taxonomic units (OTU); (2) higher alpha diversity in maternal stool; (3) dissimilarity in maternal stool OTU; (4) increased relative abundances of genus *Prevotella* in maternal stool.

#### Diet

Women in the intervention group will (1) improve their diet in accordance with the Australian dietary guidelines; (2) consume a wider variety of foods; and increase intakes of (3) fiber, (4) prebiotic foods, and (5) probiotic foods compared with the control group, and these changes will be sustained throughout pregnancy. The intervention group will reduce intakes of (6) refined processed foods, (7) saturated fat, and (8) total energy compared with the control group. 

Further secondary hypotheses for other biological outcomes (ie, short chain fatty acid [SCFA] concentration, inflammation, stress, and epigenetic regulation) and study feasibility are listed in Multimedia Appendix 1.

### Primary Outcome Measure

A between-group difference in microbial alpha diversity, measured using the Shannon Diversity Index (which accounts for species richness and evenness) at follow-up (4 weeks after birth) in the infant stool samples.

### Secondary Outcome Measures

#### Infant Microbiota

Between-group differences in other diversity measures including inverse Simpson index (a measure of richness and evenness that is less affected by rare species compared with the Shannon index) and Chao1 (measure of species richness); observed species and phylogenic diversity (measure of diversity including phylogenetic distance); the relative abundance of OTU; beta diversity using measures of between-sample dissimilarity.

#### Maternal Microbiota

Between-group differences in the relative abundance of OTU; beta diversity using measures of between-sample dissimilarity in response to dietary intake. Baseline-adjusted between-group differences in alpha diversity indices (Shannon Diversity, Inverse Simpson, and Chao1); observed species and phylogenic diversity.

#### Dietary Intake

Long-term (3-month) maternal dietary quality and variety are evaluated by applying the Dietary Guideline Index 2013 (DGI-13 scores) [23] to the validated Dietary Questionnaire for Epidemiological Studies v2 (DQES) [24], measured at baseline and 4 weeks after birth. DGI-13 scores include a total diet quality score and 13 subscores to evaluate each dietary guideline [25]. Daily macronutrients from the DQES are energy (kilojoules; kJ), protein (kJ), carbohydrate (kJ), fat (kJ), saturated fat (kJ) and fiber (grams). Time-related trends in short-term (2-week) diet quality and intakes of prebiotic and probiotic foods are evaluated using a version of the Simplified Dietary Questionnaire (SDQ) [26] modified to include prebiotic and probiotic foods. SDQ scores include a total diet quality score and comprises a dietary variety score and 9 subscores evaluating each guideline [25], along with 2 additional scores for prebiotic and probiotic food intake.

Further secondary outcome measures for other biological outcomes and study feasibility are listed in Multimedia Appendix 2.

#### Study Setting

The study is based at the Murdoch Children’s Research Institute (MCRI) at the Royal Children’s Hospital in Parkville, Melbourne, VIC.

#### Eligibility Criteria

Participants were eligible if they did not meet the exclusion criteria and could attend a Saturday workshop at the Royal Children’s Hospital between weeks 26 and 29 of gestation.

#### Exclusion Criteria

To ensure practicality, participants were excluded if they were aged under 18 years, not in control of their diet (including choice of foods purchased and meals eaten), were uncomfortable communicating in English, or resided further than 1 hour’s travel from the Hospital. To ensure suitability of the dietary intervention, participants were excluded if they had a clinically diagnosed bowel condition or were on a medically advised exclusion or restriction diet. To assess intervention efficacy, participants needed to be free of conditions that may alter their gut microbiota. Hence, participants must not have had any of the following: a body mass index of 30 or greater; diabetes mellitus (type 1, 2, or gestational diabetes); a clinical diagnosis of a current mental illness (including major depression, dysthymia, anxiety disorder, social phobia, posttraumatic stress disorder, obsessive compulsive disorder, panic disorder, an eating disorder [anorexia, bulimia, and binge-eating disorder]), psychotic disorder (schizophrenia), substance use disorder, autism disorder, attention deficit hyperactivity disorder, attention deficit disorder; or used antibiotics or probiotic supplements in the previous month; or regularly use illicit drugs.

#### Sample Size and Power

The study was powered to detect a between-group difference in alpha diversity (Shannon index) in infants. At design time in 2015, very few studies reported infant alpha diversity measured at 4 weeks, and no studies that we are aware of have ever reported differences in infant alpha diversity as a function of a maternal dietary intervention; thus, it was difficult to estimate an expected change. Instead, a clinically relevant difference in Shannon index was determined based upon the small number of case-control studies reporting differences between the Shannon index of healthy 4-week old infants to those with health problems (such as allergy) [2,4,27]. Across these studies, the mean between-group difference in Shannon index ranged between 0.2 and 0.3. Standard deviations were derived for each group ranging between 0.2 and 0.5, with 0.4 being most common. On the basis of these data, the sample size was calculated using the power.*t* test function of the R *stats* package (R Core Team, version 3.2.0). A sample of 80 mothers would provide 80% power to detect a difference in Shannon index of at least 0.25, assuming a standard deviation of 0.4 and a 2-sided type I error of 0.05. Therefore, 90 pregnant women would be recruited to participate, this permitted a loss to follow-up of 10 participants (12.5%).

### Recruitment

Melbourne-based women were recruited online or within the community (obstetric clinics, doctor’s surgeries, maternal and child health centers, childcare, playgroups, toy libraries, shopping centers, physiotherapy centers, sports centers, and radio). Online recruitment strategies included pregnancy forums, twitter, and paid Facebook advertisements that targeted Melbourne-based women aged between 18 and 40 years who met Facebook pregnancy-related demographic characteristics.

### Randomization

The randomization process used a concealed 1:1 group allocation ratio with randomly permuted block sizes to ensure allocation was unpredictable. External personnel prepared the randomization schedule and applied it to the Research Electronic Data Capture (REDCap) randomization module [28]. The study administrator used REDCap to randomly allocate each participant. After allocation, blinding was no longer possible because the team had to book study visits or a workshop.

### Participation

As a gesture of appreciation, participants received an Aus $20 grocery store gift voucher at the initial study visit. In recognition of effort, completed participants entered a raffle to win an Apple iPad, this was drawn at the end of the study. When the study results are known, participants will receive a summary of results and an invitation to attend a presentation.

### Intervention

The objectives of the dietary intervention were that participants become educated, motivated, empowered, and equipped with the skills and self-efficacy to make long-term dietary change targeting the gut microbiota. The gut microbiota were targeted as the intervention’s change mechanism, and the target behavior was *eating for the gut microbiota*. We expected that the intervention would be feasible and accepted because when asked about support preferences, pregnant women wanted nutrition education, preferably in person and individually tailored [18,29].

#### Intervention Procedures

Participants attended a dietary workshop between gestation weeks 26 and 29. Participants devised and agreed upon 3 personalized dietary goals, and they received 2 support calls to encourage adherence. Intervention procedures are detailed in Multimedia Appendix 3. For intervention fidelity, each workshop and support call followed a predefined facilitator script to ensure that all participants received the same information.

#### Dietary Recommendations

The intervention aimed to align participants’ diets to the Australian Dietary Guidelines [25], and increase intakes of fibrous plant-based foods, while reducing intakes of highly refined and processed foods. Common probiotic species (*Lactobacillus* and *Bifidobacterium*) are reported to be safe during pregnancy [30], and perinatal probiotic supplementation may increase Bifidobacterial species in infants [11]. The intervention took a sustainable, *whole of diet approach* where prebiotic- and probiotic-containing food sources were recommended to participants instead of using supplements. This *synbiotic* combination of prebiotic and probiotic foods may help to promote the growth of probiotic species [31].

#### Educational Design

The educational design used the theory of constructive alignment [32], which argues that alignment among intended learning outcomes, learning activities, and assessment is crucial for learning. Clear learning outcomes were developed for the workshop (Multimedia Appendix 4) using the Structure of Observed Learning Outcomes taxonomy [33], which allows targeting particular levels of functioning with respect to knowledge. Learning activities were designed to provide opportunities for learners to practice the particular learning outcomes; this aligns with constructive alignment’s focus on what learners do rather than on what educators do. Participants engaged in active learning tasks like problem solving, which have been shown to be more effective for learning than transmissive or *lecture* style teaching [34]. At an educational psychology level, learning activities were designed with consideration of cognitive load theory [35] to manage the demands on participants’ working memory; this was deployed through chunking content, provision of reference materials, and careful use of different media. The workshop focused on developing participants’ ability to make judgments about their diet quality. The ability to self-assess is crucial for long-term retention and application of knowledge beyond the workshop [36], as participants need to be able to judge the quality of their diet to improve it. An expert in educational design and pedagogy reviewed the workshop materials. The logic model in Multimedia Appendix 4 details how the educational design, monitoring, feedback activities, and BCTs were used in the intervention.

#### Behavior Change Techniques

To support adherence, the intervention used BCTs [21]. Behavior change is an effective method for supporting dietary adherence in community-based interventions [37-39], including among pregnant populations [40]. Successful dietary BCTs include social support [37,38], information [40], instruction [39,40], self-monitoring [39,40], self-efficacy [38], goal-setting [37,39], goal review [39], relapse prevention techniques [39], motivational interviewing [39,40], feedback provision [39], and rewards (if goals are met) [40]. Descriptions of the intervention’s use of Michie et al’s BCTs [21] are available in Multimedia Appendices 3 and 4.

### Control Group

Treatment as usual was used as an active control condition. Participants continued receiving dietary advice from a health care provider who was managing their pregnancy. The rationale for using treatment as usual was to be able to compare the intervention against standard treatment [41]. In addition, provision of a different treatment as an active control may have introduced factors that could have altered the gut microbiota or bias the results.

Participants from both groups reported on the dietary advice that they received from their health care provider. Both groups also reported on their dietary intake, including prebiotic and probiotic foods, and dietary supplements. All nonwithdrawn participants received the intervention materials in written form upon study closure after sample and data collection closed.

### Data and Sample Collection

Data were collected at 4 time points: gestation week 26, 31, 36, and 4 weeks after birth (follow-up), as detailed in Table 1. Data collection included demographic, physical health and medications, mental health and social support, diet, lifestyle, and evaluative feedback. A probiotic food and drink questionnaire was administered at all time points for the intervention group, but only at baseline and follow-up for the control group to prevent the control group from becoming aware or prompted to increase intake of probiotic foods.

The study team were trained to collect baseline and follow-up anthropometrics and biological samples. Biological samples were collected as outlined in Table 2. Participants collected a baseline stool sample during gestation week 26, and a follow-up sample from themselves and their infant 4 weeks after birth. Stool samples were stored in the domestic freezer and transported on ice to the study visit scheduled during week 26 of gestation (or before week 29). The study team collected the follow-up samples during a home visit. Samples were transported to long-term storage (−80°C) on dry ice. At the conclusion of the study, stool samples were couriered on dry ice to the Australian Genomic Research Facility (AGRF) for DNA extraction and 16S rRNA sequencing. The V3-V4 hypervariable region of the 16S rRNA gene was amplified using a forward primer, 341F, 5’-CCTAYGGGRBGCASCAG-3’ and reverse primer, 806R, 5'-GGACTACNNGGGTATCTAAT-3'. Polymerase chain reaction amplicons were generated from approximately 100 ng of extracted DNA, and purified amplicons were sequenced using Illumina MiSeq, in accordance with the manufacturer specification and AGRF protocols.

**Table 1:** Data collection schedule.

Measurement/instrument		Baseline (gestation week 26; mother)	Progress (gestation weeks 31 and 36; mother)	Follow-up (4 weeks postpartum)	
				Mother	Infant
**Demographics**					
	Demographics, socioeconomic status, ethnicity, household composition, and pets	✓	—^a^	—	—
**Physical health**					
	Medical health	✓	✓	✓	✓
	Medication and supplement use	✓	✓	✓	✓
	Oral health	✓	—	✓	—
	ROME III Diagnostic Questionnaire for Adult Functional Gastrointestinal Disorders	✓	—	✓	—
	Childbirth details	—	—	✓	
	Anthropometrics: body mass index, weight, height	✓	—	✓	✓
	Head circumference	—	—	✓	✓
**Maternal psychological well-being and relationships**					
	The Edinburgh Postnatal Depression Scale [42]	✓	—	✓	—
	Depression Anxiety and Stress Scale-21 [43]	✓	✓	✓	—
	Perceived stress scale [44]	✓	—	✓	—
	Big-5 personality scale [45]	✓	—	—	—
	Multidimensional scale of perceived social support [46]	✓	—	✓	—
	Nature relatedness scale [47]	✓	—	✓	—
**Diet**					
	Dietary Questionnaire for Epidemiological Studies (Version 2) [24]	✓	—	✓	—
	Simplified Dietary Questionnaire [26] (modified)	✓	✓	✓	—
	Probiotic Food and Drink Questionnaire	✓	✓^b^	✓	
	Infant diet	—	—	—	✓
**Lifestyle**					
	International Physical Activity Questionnaire [48]	✓	—	✓	—
	Smoking	✓	—	✓	—
**Process evaluation**					
	Workshop evaluation	—	✓^b^	—	—
	Study evaluation	—	—	✓	—
	General self-efficacy scale [49]	✓	—	✓	—
	Motivation and readiness to change [50]	✓	✓	✓	—
	Intervention personal goals	—	✓^b^	—	—

^a^Not collected.

^b^Intervention group only.

**Table 2:** Biological sample collection schedule.

Measurement		Baseline (gestation week 26; mother)	Follow-up (4 weeks postpartum)	
			Mother	Infant
**Microbiota and metabolites**				
	Stool sample	✓	✓	✓
**Stress and inflammatory markers**				
	Saliva	✓	✓	—
	Guthrie spot	—^a^	—	✓
**Epigenetic regulation**				
	Buccal cells	✓	✓	✓

^a^Not collected.

### Data Management and Access

Questionnaires were administered electronically to participants through REDCap [28], which is hosted on secure servers at MCRI. Information is kept confidential through a secure password-protected system and restrictive user-access permissions. Study team access to participant information was strictly limited to the purposes of running the study, such as organizing study visits, support calls, and recording biological sample collection. For analysis, the investigators have access to the final deidentified trial dataset.

### Monitoring

Being a community-based health intervention, minimal harms are foreseen. No independent bodies were developed for data monitoring or auditing trial conduct. Any adverse events would be reported to the Human Research Ethics Committees (HRECs) in accordance with the safety reporting policy of the HREC. SD, JMC, and FNJ oversaw the implementation of the study, and the HRECs of The Royal Children’s Hospital and Deakin University review its progress.

### Availability of Data and Materials

Data sharing is not applicable, and no data have been reported.

### Statistical Methods

The study results will be reported in accordance with the Consolidated Standards of Reporting Trials (CONSORT) guidelines [51]. Analyses will be performed according to a modified intention-to-treat principle, in which participants with at least 1 valid postbaseline follow-up are included.

#### Primary Outcome

The infant Shannon Diversity Index (collected 4 weeks after birth) will be calculated using the phyloseq R package [52]. Normal distribution will be assessed by visually inspecting quantile-quantile plots. If data are normally distributed, then an independent student *t* test will be conducted, otherwise a Wilcoxon-Mann-Whitney U test will be conducted to determine between-group differences, where statistical significance is considered at the P<.05 level [53].

#### Additional Multivariable Analysis

Additional multivariate analysis will be performed to examine potential effect modifiers (sample storage duration, birth mode, antibiotic exposure, gestational age, and mode of feeding) using Kraemer et al’s [54] approach as a guideline.

#### Secondary Outcomes

#### Gut Microbiota

Infant stool alpha diversity will be further analyzed using 4 other measures (Inverse Simpson, Chao1, phylogenic diversity, and observed species) using the same methods described for the primary outcome. All 5 alpha diversity measures will be analyzed for mothers adjusting for baseline measures. Further analyses will examine potential effect modification for storage duration, baseline, antibiotic, and medication use in accordance with Kraemer et al [54]. Between-group differences in the relative abundance of genus *Prevotella* will be analyzed using the same methods described for the primary outcome for infants and a baseline-adjusted method for the mothers.

A total of 2 beta diversity metrics will be calculated (Generalized UniFrac distances [55] and Bray-Curtis dissimilarity.) Group-based separation will be visually inspected using principal coordinates analysis and constrained ordination plots of these beta diversity metrics. Further plots will be created to inspect separation based on potential effect modifiers: mode of birth, antibiotics, sample collection week, and mode of feeding. Permutational multivariate analysis of variance (PERMANOVA) [56] with 999 permutations will be used on the beta diversity metrics to determine the statistical significance of group-based separation. In a secondary analysis, these PERMANOVA models will test for the aforementioned potential effect modifiers. Differential abundance testing with a false discovery rate correction will be performed to explore OTUs that are different between groups. The appropriate transformation and test will be determined in accordance with Weiss et al once library size is known [57]. For significant outcomes, the role of dietary change or probiotic or prebiotic supplementation in potentially mediating microbial outcomes will be examined.

#### Intervention Efficacy and Dietary Intake

Linear mixed model analyses using the generalized estimating equation (GEE) technique will be used to evaluate between-group baseline-adjusted mean differences, accounting for within participants autocorrelations across multiple time-points, for the 14 DGI-13 long-term diet measures [23], 6 macronutrient measures [24], and the 13 SDQ short-term diet measures [26]. All tests will be 2-sided, and statistical significance is considered at a P value <.05. No correction for multiple comparison will be implemented for these outcome analyses as these comparisons are *a priori* research questions with specified alternative hypotheses. Time trends in short-term diet quality and intakes of prebiotic and probiotic foods are evaluated from baseline to before birth, and baseline to after birth. For significant outcomes, the role of motivation or mental well-being in potentially mediating any impact of the intervention on dietary intake will be examined.

Analysis plans for all nonmicrobial secondary outcome measures including SCFAs, inflammation, epigenetic regulation, behavior change, well-being, feasibility, and acceptance are detailed in Multimedia Appendix 5.

### Ethics Approval and Consent to Participate

This study was approved by the Royal Children’s Hospital Human Ethics Committee on the December 17, 2015 (HREC 35200), and Deakin University Human Ethics Committee on the February 16, 2016 (DUHREC 2016-036). Current protocol:** **Version 15, November 17, 2017. Protocol modifications will be detailed in subsequent papers.

Participants provided written informed consent and may have optionally consented to (1) be contacted about future related research and (2) to have data and samples used for future ethically approved research.

## Results

The study is ongoing as results are yet to be written. The first participant was enrolled on July 28, 2016, and the final follow-up assessment was completed on October 11, 2017.

## Discussion

This is the first study to test the efficacy of an educational dietary intervention in influencing the gut microbiota of mothers and infants. This study has many strengths, including its RCT design, strict inclusion and exclusion criteria, robust intervention design, wide range of data and bio-specimen collection, and an *a priori* analysis plan.

The intervention design was particularly important because when studies depend on nutrition education to test the effects of dietary change, it can be difficult to determine whether any null results are because of participants not learning, or because of the hypothesized dietary mechanism. If participants do not learn, then they cannot be expected to change their dietary behavior. Thus, study validity is dependent, to an extent, on the quality of the nutrition education. A previous systematic review found that the use of theory was associated with nutrition education intervention success [58]; however, we draw a distinction between dominant high-level theories used in those studies (eg, social cognitive theory) and the lower level educational design theories used in this study. Higher level theories provide less of an evidence base for practical matters, such as the translation of study objectives to intended learning outcomes, mapping outcomes to specific activities, and the selection of appropriate media or designing PowerPoint slides. Lower level educational design considerations are important because they are influential in how well participants learn. The intervention design is a key strength of this study with its clear alignment among the target behaviors, intended learning outcomes, learning activities, BCTs, and participant self-monitoring.

Regardless of a participant’s baseline diet quality, we expect that the intervention will be effective in increasing average intakes of fiber, and prebiotic, and probiotic foods. These foods were specifically targeted in the intervention, while the control group was unaware of the interest in these prebiotic and probiotic foods. We anticipate that the diversity of the prenatal gut microbiota is stable and will respond to this dietary change. DiGiulio et al demonstrate that stool alpha diversity is stable from week-to-week during pregnancy and the postpartum period [59]. Elsewhere, Koren et al report that there is significant instability during pregnancy [60]. However, samples were only collected at 3 time points (not weekly), and critically, a subset of participants may have been involved in a dietary intervention consuming probiotics [61]. In nonpregnant adults, gut microbiota respond to short-term dietary intake within 24 hours [62,63]. Hence, dietary change needed to be sustained through to follow-up when the final stool sample was collected. We addressed this in our intervention design through the use of BCTs [21,39], and during the last support call (before birth), we discussed how each participant plans to sustain their dietary goals after birth (Multimedia Appendix 3).

The study is powered to test for an overall effect of the intervention. The overall intervention effect will be unbiased on the basis that the study has an RCT design, where we expect that potential effect modifiers or confounders (both measured and more importantly unmeasured) will be balanced out between groups. The study will not be underpowered unless there is strong heterogeneity because of a potential effect modifier. Based upon the population rates, we expect the majority of births to be vaginal, and majority of infants will be breast fed (67% vaginal birth, 33% cesarean [64], with 74.6% breastfeeding at 1 month [65]). The study may not have power to detect the role of effect modifiers, but this was not the main study aim. Our sample size calculation was based upon an estimated standard deviation of 0.4, we recently reassessed the accuracy of this estimate using recently available 16S data for 144 4-week old infants from the INFANTMET cohort [66]. We analyzed these data and arrived at a standard deviation of 0.317; this is lower than our original estimate, indicating that our sample size calculation may be conservative.

Given that this is the first study to measure changes in Shannon index of 4-week old infants as a function of a perinatal dietary intervention, we could not base the effect size upon established dietary intervention data. Instead, we used a clinically meaningful difference in Shannon index, by basing the calculation on detecting a difference in Shannon index as small as 0.25. This represents the mean between-group difference in Shannon index between allergy case and controls at 4 weeks across 3 studies [2,4,27]. If this study is efficacious, then our use of a clinically meaningful difference may assist in interpreting and translating the results. We urgently need data from human experimental studies (such as this study) to inform similar interventions. Without pre-existing data, it is difficult to estimate whether the selected effect size is too optimistic. Importantly, this study will generate the data needed to inform power calculations for future perinatal dietary intervention studies.

### Conclusions

To our knowledge, there are currently no human trials testing the hypothesis that the diversity and composition of the infant gut microbiota is modifiable through the perinatal diet. Animal studies implicate poor maternal diets (high intakes of fat or low fiber) in the disturbance of gut microbiota in offspring [8,9]. Experimental studies are needed to determine whether this holds in humans. This is particularly important because diet quality during pregnancy appears to be poor, with many women failing to meet recommendations for fiber and energy and exceeding the recommendation for fat intake [17]. Data arising from this study may inform future interventions aiming to target the composition of the gut microbiota in early life. The results of this study may also be used to inform clinical and public health recommendations supporting the gut microbiota in early life.

## Appendix

Multimedia Appendix 1 Secondary hypotheses.

Multimedia Appendix 2 Secondary outcome measures.

Multimedia Appendix 3 Intervention procedures and behavior change technique usage.

Multimedia Appendix 4 Intervention logic model.

Multimedia Appendix 5 Statistical methods for secondary outcomes.

## Abbreviations

BCT: behavior change techniques
DGI-13: Dietary Guideline Index 2013
DQES: Dietary Questionnaire for Epidemiological Studies
HPHK: healthy parents, healthy kids
kJ: kilojoules
MCRI: Murdoch Children’s Research Institute
OTU: operational taxonomic units
PERMANOVA: permutational multivariate analysis of variance
RCT: randomized controlled trial
REDCap: research electronic data capture
SCFA: short chain fatty acid
SDQ: Simplified Dietary Questionnaire
