# Supplementary material for: Targeting the Infant Gut Microbiota Through a Perinatal Educational Dietary Intervention: Protocol for a Randomized Controlled Trial
Source: JMIR Res Protoc. 2019 Oct 21;8(10):e14771. doi: 10.2196/14771 (PMC6914305; doi:10.2196/14771)
Supplement: Multimedia Appendix 1 [file resprot_v8i10e14771_app1.pdf]

Multimedia Appendix 1. Secondary Hypotheses

Infant Biological Outcomes

Using samples collected at follow-up, compared to the control group, infants in the intervention group will have: (1) a greater concentration of stool short chain fatty acids (SCFA); (2) lower levels of biomarkers of stress and inflammation in resuspended dry blood spots; (3) differences in epigenetic regulation (differentially methylated CpGs) measured using buccal cells; (4) DNA methylation differences in CpGs in free fatty acid receptor genes *FFAR2* and *FFAR3*.

Maternal Biological Outcomes

Using samples collected at follow-up, compared to the control group, mothers in the intervention group will have: (1) a greater concentration of stool short chain fatty acids; (2) lower levels of biomarkers of stress and inflammation measured in saliva samples; (3) differences in epigenetic regulation measured using buccal cells; (4) DNA methylation differences in CpGs in free fatty acid receptor genes *FFAR2* and *FFAR3*.

Process Evaluation, Feasibility and Acceptance

The study implementation will be feasible and the procedures will be acceptable to participants.

Secondary outcome measures for the gut microbiota and maternal diet are detailed in the study protocol.
