# Supplementary material for: Targeting the Infant Gut Microbiota Through a Perinatal Educational Dietary Intervention: Protocol for a Randomized Controlled Trial
Source: JMIR Res Protoc. 2019 Oct 21;8(10):e14771. doi: 10.2196/14771 (PMC6914305; doi:10.2196/14771)
Supplement: Multimedia Appendix 3 [file resprot_v8i10e14771_app3.pdf]

Multimedia Appendix 3. Intervention Procedures and Behavior Change Technique Usage

At the Intervention Workshop

The intervention procedures were mapped to Michie et al's BCTs [1] as indicated in brackets. To reduce negative emotions around altering dietary intake (including restricting discretionary food intake), the workshop positively reframed *diet* by promoting potential benefits to health and the gut (13.2, 11.2). To inspire a willingness and commitment to make dietary improvement, participants were asked to reflect on their diet and draw a dietary pyramid showing the food groups that they eat from, from most to least. Participants were told about the importance of diet for physical health and mental wellbeing (5.1, 5.6). The relationships between diet quality and mental wellbeing were discussed with reference to landmark studies to reinforce credibility (9.1). Participants were asked to reflect on their dietary pyramid (drawn earlier), compare it against the dietary guidelines [2,3] and identify discrepancies between the two (1.6). They discussed what they would need to do to meet the dietary guidelines, and reflected on their willingness change, and then discussed three personalised dietary goals to aim for (1.1).

Participants were taught to use a plate-based guide for pregnancy [4], which was to be taken home to prompt healthy meal planning (4.1, 7.1). Participants practiced activities to align the previous night's dinner to the plate-based dietary guide for pregnancy [4] to ensure that they could apportion a meal for diet

quality and quantity (6.1, 8.1, 8.2). They were given a take-home list of prebiotic [5] and probiotic-containing foods for targeting the gut microbiota, and were shown how to use this resource as a shopping list (to restructure the foods kept at home), and for meal and snack planning (4.1, 7.1, 12.1). In another activity, the previous night's dinner was further augmented to include the prebiotic and probiotic ingredients on the list (6.1, 8.1, 8.3). As a group, participants problem-solved to find solutions to barriers preventing them from eating as practised in the activities (1.2). Participants were asked to continue considering whether they would like to eat this way and devise their implementation intentions for what they would need to do to achieve this (1.4). Lunch and morning tea were provided to demonstrate how to prepare healthy meals and how to incorporate certain foods to target the gut microbiota into everyday meals (4.1, 6.1, 8.2). Participants were taught how to eat according to the Australian food safety recommendations for pregnancy [4,6] (4.1). Participants were shown and provided with a link to videos, recipes and credible information about high fibre and prebiotic diets for use at home [5] (4.1, 9.1). At the end of the workshop, participants were asked to set a minimum of three SMART (specific, measurable, action oriented, realistic and time-based) dietary goals outlining their implementation intentions to target their gut microbiota (1.1, 1.4). Goals were committed to through a written behavioral change contract (1.8, 1.9), which included a description for self-reward if goals were met (10.3, 10.7, 10.9).

At Home

All participants received two support phone calls, one at week 31 and the other at week 36 of gestation. Calls followed a set of structured questions delivered by

a trained facilitator taking notes (3.1). Support calls were designed to assist with adherence to healthy eating through self-monitoring and re-evaluating goals. Participants were asked to list their SMART (specific, measurable, action oriented, realistic and time-based) dietary goals, and they evaluated how they felt they had gone with meeting their goals (1.6, 2.3). The facilitator focused on the successes (15.3) and asked participants to talk about barriers to meeting their goals and to suggest some ways these could be overcome (1.2). The facilitator discussed possible solutions to barriers if participants were unable to identify solutions, such as methods of restructuring the physical environment (2.2, 3.2, 12.1). Participants were provided with an opportunity to ask questions about their diet and receive feedback on their progress towards meeting their dietary goals (2.2). The facilitator tailored feedback based on the participant's needs, within the context of the intended intervention learning outcomes. The aim of this feedback was to refine the participant's self-assessment of both their diet, and their progress towards meeting their goals. Participants were asked whether they would like to change their SMART goals or recommit to the same ones (1.5, 1.9). The last support call included discussion and support for the continued achievement of dietary goals during the postnatal period (1.2).

References

1. Michie S, Richardson M, Johnston M, Abraham C, Francis J, Hardeman W, Eccles MP, Cane J, Wood CE. The behavior change technique taxonomy (v1) of 93 hierarchically clustered techniques: Building an international consensus for the reporting of behavior change interventions. Ann Behav Med 2013;46(1):81–95. PMID:23512568

2. Nutrition Australia. Healthy Eating Pyramid [Internet]. Nutrition Australia;

2015 [cited 2015 Jun 10]. Available from: http://www.nutritionaustralia.org/national/resource/healthy-eating-pyramid

3. National Health and Medical Research Council. Australian Dietary Guidelines [Internet]. Canberra; 2013. Available from: https://www.eatforhealth.gov.au/sites/default/files/files/the_guidelines/n55_australian_dietary_guidelines.pdf

4. Oregon Health & Science University. My Pregnancy Plate [Internet]. Oregon Heal Sci Univ. 2015 [cited 2015 Jul 6]. Available from: https://www.ohsu.edu/xd/health/services/women/services/other-services/upload/CWH-3051517-Pregnancy-Plate-FLY-WEB.pdf

5. Monash Department of Gastroenterology. High fibre, high prebiotic diet for healthy individuals [Internet]. Monash Univ. 2015 [cited 2015 May 1]. Available from: https://www.monash.edu/medicine/ccs/gastroenterology/prebiotic

6. National Health and Medical Research Council. Healthy eating during your pregnancy, advice on eating for you and your baby [Internet]. Canberra: Australian Government, National Health and Medical Research Council; 2013. Available from: https://www.eatforhealth.gov.au/sites/default/files/content/The Guidelines/n55h_healthy_eating_during_pregnancy.pdf
