# Supplementary material for: Targeting the Infant Gut Microbiota Through a Perinatal Educational Dietary Intervention: Protocol for a Randomized Controlled Trial
Source: JMIR Res Protoc. 2019 Oct 21;8(10):e14771. doi: 10.2196/14771 (PMC6914305; doi:10.2196/14771)
Supplement: Multimedia Appendix 4 [file resprot_v8i10e14771_app4.pdf]

Multimedia Appendix 4. Intervention Logic Model

Table A4.1: Intervention logic model describing the design features used to meet the intervention objectives

**Workshop (Gestation week 26-29)**	
**Transmission teaching component, 1.5 hours**	
Instructional design	
Objectives:	To communicate foundation knowledge about the importance of a healthy prenatal diet for maternal and infant health and the gut.<br>To explain what a healthy diet comprises.<br>To inspire participants to want to act to improve their diet.
Learning outcomes:	At the end of the workshop, participants should be able to:<br>1. Describe the basic effects of healthy and poor eating on the general and mental health outcomes of women and children<br>2. Describe the influence of diet quality on gut microbiota, role of fibre, prebiotic and probiotic-containing foods<br>3. Identify the foods groups in the healthy eating guidelines<br>4. Relate the proportions in which each food group should be consumed<br>5. Reflect on own willingness to change eating behavior
Teaching resource:	Scripted presentation
Use of Behavior Change Techniques	
Target Behaviors:	Knowledge building; Motivation to change
BCTs [1]:	Framing / reframing (13.2); Reduce negative emotions (11.2); Information about emotional consequences (5.6); Information about health consequences (5.1); Credible sources (9.1)
**Active learning component, 1.5 hours**	
Instructional design	
Objective:	To equip participants with practical skills, resources and confidence needed for implementing healthy dietary change at home.
Learning outcomes:	At the end of the workshop, participants can:<br>6. Outline the steps needed to align own diet with dietary guidelines<br>7. Align the composition of their meal with a take home plate-based guide to assist with meeting the dietary guidelines<br>8. Apply food safety principles throughout pregnancy<br>9. Identify the prebiotic and probiotic-containing foods already consumed<br>10. Augment daily meals and recipes to include prebiotic and probiotic-containing food ingredients<br>11. Problem-solve to overcome social and environmental challenges/ barriers to adopting a healthy diet<br>12. Develop strategies to incorporate high fibre, prebiotic and probiotic foods into daily meals<br>13. Plan shopping list and meals with reference to take home resources

	so that participants have access to healthy foods and can include foods targeting the gut microbiota
Teaching resources:	Scripted presentation. Take home: Pregnancy plate based guide[2], Nutrition Australia food pyramid [3] and dietary guidelines [4], Prebiotic [5] and probiotic-containing food list, URL to Monash University website for high fibre and prebiotic diet videos, recipes and information [5]
Use of behavior change techniques	
Target behaviors:	Healthy eating; Augmenting meals to include foods for gut health<br>Skill building; Self-efficacy; Overcoming perceived barriers; Problem solving
BCTs [1]:	Discrepancy between current behavior and goal standard (1.6); Instruction on how to perform a behavior (4.1); Behavioral rehearsal/practice (8.1); Behavior substitution (8.2); Problem solving/coping planning (1.2); Identification of self as role model (13.1) Social support (general) (3.1); Demonstration of the behavior (6.1); Adding objects to the environment (12.5); Prompts/cues (7.1); Habit formation (8.3); Restructuring the physical environment (12.1)
**Action planning, 20 minutes**	
Instructional design	
Objective	To ensure that participants have a written behavior change contract with a set of personalised SMART goals to meet for the duration of the study.
Learning outcomes:	14. Set and review own dietary goals
Resources:	Take home resource: Behavior change contract
Use of behavior change techniques	
Target behaviors:	Action planning and goal setting for dietary improvement
BCTs [1]:	Goal setting (behavior) (1.1); Behavioral contract (1.8); Action planning (including implementation intentions) (1.4); Commitment (1.9) Non-specific reward (10.3); Self incentive (10.7); Self-reward (10.9)
**Morning tea and Lunch, 40 minutes**	
Objective:	To demonstrate how to incorporate foods considered good for gut health into everyday meals.
Monitor learning outcomes:	7. Align the composition of their meal with a take home plate-based guide to assist with meeting the dietary guidelines<br>10. Augment daily meals and recipes to include prebiotic and probiotic-containing food ingredients
Resources:	Serving equipment, nuts, seasonal fruits, chopped vegetables, hummus, dip, salad, hard cheese, felafel, rye or wholemeal bread, water
Use of behavior change techniques	
Target behaviors:	Healthy eating; Augmenting meals to include foods for gut health
BCTs [1]:	Instruction on how to perform a behavior (4.1); Modelling of the behavior (6.1)
**Monitoring activities (Gestation weeks 31 and 36), 20 minutes**	
Dietary intake questionnaire, 15 minutes	
Resources:	Redcap administered online Simple Dietary Questionnaire [6]
Use of behavior change techniques	

Target behavior:	Healthy Eating
BCTs [1]:	Self-monitoring of behavior (2.3)
Motivation and readiness to change questionnaire, 5 minutes	
Resources:	Redcap administered online Motivation and Readiness to change questionnaire [7]
Use of behavior change techniques	
Target behavior:	Motivation
BCTs [1]:	Self-monitoring of behavior (2.3)
Support telephone call with scripted questions, 10 – 20 minutes	
Instructional design	
Monitoring and feedback for the following learning outcomes (set above):	5. Reflect on own willingness to change eating behavior<br>6. Outline the steps needed to align own diet with dietary guidelines<br>7. Align the composition of their meal with a take home plate- based guide to assist with meeting the dietary guidelines<br>9. Identify the prebiotic and probiotic-containing foods consumed<br>10. Augment daily meals and recipes to include prebiotic and probiotic-containing food ingredients<br>11. Problem-solve to overcome social and environmental challenges/ barriers to adopting a healthy diet<br>12. Develop strategies to incorporate high fibre, prebiotic and probiotic foods into daily meals<br>13. Plan shopping list and meals with reference to take home resources so that participants have access to healthy foods and can include foods targeting the gut microbiota<br>14. Set and review own dietary goals
Resources:	Participant: Behavior change contract and workshop take home resources<br>Interviewer: Script for support call
Use of behavior change techniques	
Target behavior:	Healthy eating; self-efficacy; problem solving
BCTs [1]:	Review behavior goal(s) (1.5); Self-monitoring of behavior (2.3)<br>Focus on past success (15.3); Goal setting (behavior) (1.1); Problem solving/coping planning (1.2); Commitment (1.9); Behavioral contract (1.8); Discrepancy between current behavior and goal standard (1.6); Restructuring the physical environment (12.1); Social support unspecified (3.1); Social support (practical) (3.2); Feedback on behavior (2.2)

Numbers within brackets refer BCTs in the Michie et al Behavior Change Technique Taxonomy [1]. BCT, Behavior Change Technique.

References

[1] Michie S, Richardson M, Johnston M, Abraham C, Francis J, Hardeman W, Eccles MP, Cane J, Wood CE. The behavior change technique taxonomy (v1) of 93 hierarchically clustered techniques: Building an international consensus for the reporting of behavior change interventions. Ann Behav Med 2013;46(1):81–95. PMID:23512568

[2] Oregon Health & Science University. My Pregnancy Plate [Internet]. Oregon Heal Sci Univ. 2015 [cited 2015 Jul 6]. Available from: https://www.ohsu.edu/xd/health/services/women/services/other-services/upload/CWH-3051517-Pregnancy-Plate-FLY-WEB.pdf

[3] Nutrition Australia. Healthy Eating Pyramid [Internet]. Nutrition Australia; 2015 [cited 2015 Jun 10]. Available from: http://www.nutritionaustralia.org/national/resource/healthy-eating-pyramid

[4] National Health and Medical Research Council. Healthy eating during your pregnancy, advice on eating for you and your baby [Internet]. Canberra: Australian Government, National Health and Medical Research Council; 2013. Available from: https://www.eatforhealth.gov.au/sites/default/files/content/The Guidelines/n55h_healthy_eating_during_pregnancy.pdf

[5] Monash Department of Gastroenterology. High fibre, high prebiotic diet for healthy individuals [Internet]. Monash Univ. 2015 [cited 2015 May 1]. Available from: https://www.monash.edu/medicine/ccs/gastroenterology/prebiotic

[6] Parletta N, Zarnowiecki D, Cho J, Wilson A, Bogomolova S, Villani A, Itsiopoulos C, Niyonsenga T, Blunden S, Meyer B, Segal L, Baune BT, O'Dea K. A Mediterranean-style dietary intervention supplemented with fish oil improves diet quality and mental health in people with depression: A randomized controlled trial (HELFIMED). Nutr Neurosci 2019; 22(7):474-487. PMID: 29215971

[7] Skouteris H, McCabe M, Milgrom J, Kent B, Bruce LJ, Mihalopoulos C, Herring SJ, Barnett M, Patterson D, Teale G, Gale J. Protocol for a randomized controlled trial of a specialized health coaching intervention to prevent excessive gestational weight gain and postpartum weight retention in women: the HIPP study. BMC Public Health 2012; 12:78. PMID: 22272935
