# Supplementary material for: Targeting the Infant Gut Microbiota Through a Perinatal Educational Dietary Intervention: Protocol for a Randomized Controlled Trial
Source: JMIR Res Protoc. 2019 Oct 21;8(10):e14771. doi: 10.2196/14771 (PMC6914305; doi:10.2196/14771)
Supplement: Multimedia Appendix 5 [file resprot_v8i10e14771_app5.pdf]

Multimedia Appendix 5. Statistical Methods for Secondary Outcomes

Short Chain Fatty Acid and Inflammatory Outcomes

For the SCFA and inflammatory outcome measures, between-group mean comparison will be performed using a one-way analysis of variance if data are normally distributed, otherwise the Mann–Whitney U-test and the Wilcoxon signed-rank test will be used.

Epigenetic Outcomes

Genome-wide data analysis will be conducted using the Maksimovic et al. analysis pipeline, which includes quality control, normalization, filtering differential methylation analysis adjusted for multiple testing [1,2]. Removing Unwanted Variation is used as a method to identify CpGs that are differentially methylated accounting for unwanted technical and biological variation [1]. Biological pathways that are related to the top differentially methylated CpGs will be interrogated. Further separate gene specific between-group analysis will be performed for CpGs in *FFAR2* and *FFAR3* using multivariate regression models. These models will also examine potential effect modification for sex, and will be adjusted for multiple testing.

Maternal Wellbeing

Generalized Estimation Equation models will be used to evaluate between-group, baseline-adjusted mean differences in symptoms of depression, stress or anxiety, accounting for within participants autocorrelations, across multiple

time-points. Statistical significance will be considered at $P < .05$.

Behavior Change

Individual dietary goals from behavior change contracts will be reviewed against the Simplified Dietary Questionnaire [3] at each time-point to determine the extent to which goals were complied with. To explain the reasons why dietary goals were or were not met, two investigators will perform qualitative thematic analysis on the facilitator notes made during the dietary support phone calls.

Feasibility and acceptance

Study feasibility will be evaluated using rates for: study visit attendance; recruitment and withdrawal; sample and data missingness. *Study acceptance* will be evaluated using qualitative study evaluation feedback. The *intervention implementation* and its *acceptance* will be evaluated using: attendance rate; intervention goals compliance, and qualitative workshop evaluation feedback including participant perceived value.

References

1. Maksimovic J, Gagnon-Bartsch JA, Speed TP, Oshlack A. Removing unwanted variation in a differential methylation analysis of Illumina Human Methylation 450 array data. Nucleic Acids Res 2015;43(16):1–14. PMID:25990733
2. Maksimovic J, Phipson B, Oshlack A. A cross-package Bioconductor workflow for analysing methylation array data [version 3; referees: 4 approved]. F1000Research 2017;5(1281):1–52. PMID:27347385
3. Parletta N, Zarnowiecki D, Cho J, Wilson A, Bogomolova S, Villani A,

Itsiopoulos C, Niyonsenga T, Blunden S, Meyer B, Segal L, Baune BT, O'Dea K. A Mediterranean-style dietary intervention supplemented with fish oil improves diet quality and mental health in people with depression: A randomized controlled trial (HELFIMED). Nutr Neurosci 2019; 22(7):474-487. PMID:29215971
